# Multi-Organ Comparative Transcriptomic Study on Short-Term and Long-Term Hypoxia Adaptation in Cattle

**DOI:** 10.3390/biology15171474

**Published:** 2026-09-01

**Authors:** Li Zhu, Hao Zeng, Zhuzha Basang, Quji Suolang, Qiang Jiang

**Affiliations:** 1 Institute of Animal Science and Veterinary Medicine, Shandong Academy of Agricultural Sciences, Ji’nan 250100, China; zhuli18328815855@163.com (L.Z.); zenghao93@126.com (H.Z.); 2 State Key Laboratory of Animal Biotech Breeding, China Agricultural University, Beijing 100193, China; 3 Faculty of Animal Science and Technology, Yunnan Agricultural University, Kunming 650201, China; 4 Institute of Animal Science and Veterinary Medicine, Tibet Academy of Agricultural and Animal Husbandry Sciences, Lhasa 850000, China; bsangzhuzha2024@163.com (Z.B.); jqtaray12307@163.com (Q.S.)

**Keywords:** cattle, hypoxia, acclimatization, adaptation

## Abstract

Highland Tibetan cattle have evolved unique hypoxic adaptation capabilities from their lowland ancestors; however, the mechanisms that distinguish short-term acclimatization from long-term genetic adaptation remain poorly understood. In this study, we integrated hematological assays and multi-organ transcriptomic analyses across three groups: highland Tibetan cattle (long-term adaptation), highland-acclimatized Holsteins (short-term acclimatization), and lowland Holsteins (controls). Hematologically, highland-acclimatized Holsteins displayed elevated red blood cell count, hemoglobin level, and blood viscosity, consistent with compensatory responses to hypoxia. In contrast, Tibetan cattle maintained lower and more stable hematological parameters, reflecting a blunted adaptive strategy shaped by long-term natural selection. Transcriptomic analysis revealed tissue-specific response to hypoxia: the heart underwent metabolic reprogramming, the liver shifted from metabolic compensation toward immune homeostasis, and the lung exhibited limited transcriptional changes associated with enhanced oxygen exchange efficiency. Pathway analysis and immunohistochemistry implicated *GNB1* in PI3K–AKT-associated angiogenic responses to hypoxia. Furthermore, hepatic *SLAMF7* upregulation and recombinant SLAMF7 activity against *S. aureus* and *E. coli* suggested a potential role in long-term immune resilience. Collectively, our findings clarify the dynamic transition from short-term physiological stress responses to long-term genetic adaptation in cattle under high-altitude hypoxic conditions, providing novel insights into the hypoxic adaptation mechanisms of ruminants.

## Simple Summary

Lowland cattle introduced to high altitudes must rapidly adjust to oxygen shortage, whereas Tibetan cattle have adapted over many generations. We compared lowland Holsteins, highland-acclimatized Holsteins, and native Tibetan cattle. Highland-acclimatized Holsteins showed increased RBC, HGB, and blood-viscosity-related measures, indicating enhanced oxygen transport but possible circulatory costs. Tibetan cattle showed a more restrained blood response. Gene-expression analyses of the heart, liver, and lung revealed organ-specific responses involving energy metabolism, immune regulation, and cellular maintenance. *GNB1* and *SLAMF7* were identified as candidate genes associated with vascular and immune responses, respectively. These findings show that short-term acclimatization and long-term adaptation involve distinct physiological and molecular strategies and provide useful information for breeding and managing cattle in high-altitude regions.

## 1. Introduction

High-altitude environments—characterized by reduced pressure of oxygen, decreased ambient temperature, and elevated ultraviolet radiation—present substantial challenges to organism habitation. To adapt to such extreme environmental conditions, acclimatized newcomers undergo physiological modifications to restore homeostasis, such as regulated blood physiology, remodeled energy metabolism, and enhanced pulmonary oxygenation capacity [1]. For non-adapted low-altitude cattle (e.g., Holstein cattle), exposure to hypoxia environment triggers a cascade of effects, including diminished blood oxygen saturation, cardiopulmonary edema, and metabolic dysfunction. In contrast, native highlanders (e.g., Tibetan cattle) have evolved genetic adaptations over thousands of years, improving their viability and reproductive success [2,3,4,5,6,7]. There relatively blunted erythropoietic response may prevent excessive erythrocytosis and associated hyperviscosity, thereby reducing circulatory burden and preserving tissue perfusion during chronic hypoxia. These two distinct adaptive strategies are called short-term acclimatization (STA) and long-term adaptation (LTA). Notably, certain STA-mediated changes that confer short-term advantages can exhibit detrimental long-term effects. For instance, elevated hemoglobin concentrations enhance oxygen-carrying capacity in the short term, yet they increase the risk of chronic mountain sickness due to their association with heightened blood viscosity [8,9]. Investigations into the two distinct adaptive strategies—short-term acclimatization (STA) and long-term adaptation (LTA)—facilitate the recognition of diversity insights into high-altitude adaptation, thereby advancing the understanding of intervention strategies for altitude- or hypoxia-related illnesses.

Transcriptomic studies have revealed that the heart and lungs are key organs exhibiting adaptive transcriptional changes in yaks at different altitudes. Notably, in lung tissue, 90% of the differentially expressed genes (DEGs) showed a nonlinear relationship with altitude [10]. Another study reported that DEGs identified in the lungs of yaks from different altitudes were primarily enriched in immune response and cell cycle pathways, whereas DEGs between yaks and low-altitude Zaosheng cattle were mainly enriched in long-chain fatty acid metabolism and protein processing [11]. Beyond the heart and lungs, brain tissue of yaks showed more genes enriched in hypoxia-adaptive pathways compared to Sanjiang cattle, a low-altitude breed, with the cerebellum being particularly sensitive to hypoxia. RNA-seq analysis of lung type II cells from Tibetan and Landrace pigs revealed that DEGs were predominantly involved in glycolysis, aldosterone synthesis, and secretion, potentially explaining the superior hypoxic adaptation of Tibetan pigs [12]. Furthermore, DEGs in heart tissues between Tibetan and Yorkshire pigs were mainly associated with HIF signaling and cardiovascular development pathways, while those in lung tissues were linked to HIF signaling, immune response, gas transport, and vascular development [13]. A comparative transcriptomic study of the chorioallantois membrane in Tibetan chicken and low-altitude Huaixiang chicken embryos showed that DEGs were enriched in HIF signaling, angiogenesis and morphogenesis, blood circulation, and the renin–angiotensin system [14]. RNA-seq analysis of heart tissues from Tibetan sheep at different altitudes indicated that DEGs were primarily associated with the PI3K-Akt, Wnt, and PPAR signaling pathways and involved in regulating mitochondrial dynamics and function [15]. However, conventional binary comparisons between low- and high-altitude populations cannot distinguish acclimatization-induced physiological responses from long-term adaptive changes.

In this study, to comprehensively characterize the molecular and phenotypic changes underlying short-term hypoxic acclimatization and long-term hypoxic adaptation, we integrated hematological physiological assays, hemorheological measurements, and multi-organ transcriptomic profiling to identify key genetic drivers of hypoxia adaptation. Specifically, we aimed to assess the dynamic regulatory patterns of bovine gene expression signatures associated with acclimatization and adaptation while clarifying the corresponding phenotypic plasticity and stable adaptive traits at the hematological and hemorheological levels. By revealing the key molecular mechanisms of plateau adaptation, this research aims to provide a theoretical foundation for the selection and improvement of livestock breeds with enhanced tolerance to high-altitude environments.

## 2. Materials and Methods

### 2.1. Animal Rearing and Sample Preparation

Three experimental groups were designed: two native groups and one immigrant group. Specifically, for the native groups, we reared lowland cattle (Holstein breed) in Jinan (36°40′ N, 117°01′ E, altitude 100 m) and highland cattle (Tibetan breed) in Lhasa (29°42′ E, 91°65′ N, altitude 3500 m), China. For the immigrant group, we raised lowland cattle (Holstein breed) in Lhasa (29°42′ E, 91°65′ N, altitude 3500 m). All three groups of cattle were raised on standardized farms and fed identical total mixed ration (TMR) diets supplied by New Hope Liuhe Co., Ltd. (Chengdu, China), with consistent feeding amounts and unlimited clean drinking water. Anticoagulant whole blood was collected from healthy adult female cows aged 2–4 years in a resting state to perform blood biochemistry and hemorheology tests. The tissue samples were obtained from commercially operated slaughterhouses in Lhasa and Jinan, China. Prior to sample collection, we obtained written informed consent from the management of the slaughterhouses, authorizing us to collect residual tissue samples (after routine slaughter for commercial purposes) for experimental use. The tissue samples were frozen in liquid nitrogen and stored in 10% neutral formalin solution (Servicebio, Wuhan, China). The procedures for animal care were approved by the Animal Welfare Committee of Shandong Academy of Agricultural Sciences, and all experiments were conducted in accordance with approved relevant guidelines and regulations.

### 2.2. RNA Isolation and Sequencing

Three oxygen-sensitive tissues (heart, lung, and liver) were collected from each of the 12 samples for RNA sequencing. The standard TRIzol method (Invitrogen, Carlsbad, CA, USA) was employed for the isolation of total RNA from these tissues. Library preparation and RNA sequencing were performed by Berry Genomics Co., Ltd. (Beijing, China) using an illumina HiSeq 4000 platform (Illumina, San Diego, CA, USA). A total of 36 libraries were constructed, with an average sequencing depthof > 10×.

### 2.3. Mapping and Annotation of Sequencing Reads

Raw RNA-seq reads were filtering by removing the following type of reads: (1) more than half of the bases were fewer than 10; (2) contained more than two Ns; and (3) fewer than 20 in length. After removal of the adapters, the remaining clean reads were aligned to the reference genome (ARS-UCD1.2) (http://ftp.ensembl.org/pub/release-107/fasta/bos_taurus/dna) (accessed on 12 July 2025) HISAT2 v2.0.4 (http://www.ccb.jhu.edu/software/hisat) (accessed on 12 July 2025). Then, gene expression was estimated using edgeR (v3.16.5).

### 2.4. Functional Enrichment Analysis

Upregulated and downregulated DEGs were classified by GO and KEGG using DAVID online software (https://davidbioinformatics.nih.gov/) (accessed on 25 July 2025). The GO terms used were LC, CC, and MF, and KEGG pathways with *p*-values < 0.01 were considered extremely significantly enriched.

### 2.5. Reverse-Transcription Quantitative PCR Validation

Eight genes were selected for RT-qPCR validation: *GNB1*, *MAOB*, and *PPARGC1A* in the heart; *RGCC* and *ADAMTSL2* in the lung; and *SLAMF7*, *BHMT*, and *CFI* in the liver. Total RNA from the same biological samples used for RNA-seq was reverse-transcribed using a PrimeScript RT reagent kit with gDNA eraser (Takara Bio, Kusatsu, Shiga, Japan). RT-qPCR was performed using TB Green Premix Ex Taq II (Takara Bio, Kusatsu, Shiga, Japan) on a QuantStudio 5 real-time PCR system (Applied Biosystems, Foster City, CA, USA). The amplification program consisted of 95 °C for 30 s, followed by 40 cycles of 95 °C for 5 s and 60 °C for 30 s. RPLP0 was used as the internal reference, and each sample was analyzed in triplicate. Relative expression was calculated using the 2^−ΔΔCt^ method, with the L group as the calibrator. Primer sequences are listed in Supplementary Table S3.

### 2.6. Weighted Gene Co-Expression Network Analysis (WGCNA)

WGCNA was conducted to identify gene modules associated with hypoxic response. The WGCNA (v1.73) package in R (v4.4.3) was utilized to construct a gene co-expression network, and key modules linked to hypoxia were identified for further exploration. This analysis facilitated the understanding of gene interactions and regulatory pathways involved in the cellular response to hypoxic stress.

### 2.7. Immunohistochemistry

GNB1 protein expression was examined in 5 μm paraffin-embedded sections of the heart, liver, and lung. Slides were incubated with 3% H_2_O_2_ (Servicebio, Wuhan, China) to quench endogenous peroxidase activity and then subjeted to antigen retrieval in citrate buffer (Servicebio, Wuhan, China) for 15 min. After washing with PBS (Servicebio, Wuhan, China), the sections were blocked with normal goat serum and incubated overnight at 4 °C with a rabbit polyclonal anti-GNB1 antibody (Proteintech, Wuhan, China; catalog no. 10247-2-AP; 1:100). After PBS washing, the sections were incubated with a biotinylated goat anti-rabbit IgG secondary antibody (Boster Biological Technology, Wuhan, China) at 37 °C for 30 min, washed and further incubated with a rabbit streptavidin–biotin complex (SABC) kit (Boster Biological Technology, Wuhan, China) at 37 °C for 30 min. Immunoreactivity was visualized using 0.025% 3,3-diaminobenzidine (Biogenex, San Ramon, CA, USA), and the sections were counterstained with Mayer’s hematoxylin. Four fields per section were randomly imaged at ×400 magnification using a Leica light microscope (Leica Microsystems GmbH, Wetzlar, Germany). Image analysis was performed using Image-Pro Plus v6.0 (Media Cybernetics, Rockville, MD, USA) to determine the brown-yellow particles of mean cumulative optical density (integrated optical density, IOD).

### 2.8. Protein Analysis

For SDS-PAGE, samples were run on a 12% polyacrylamide gel (Bio-Rad Laboratories, Hercules, CA, USA) and stained with Coomassie brilliant blue (Servicebio, Wuhan, China). For Western blot analysis, proteins from SDS gel were transferred to a PVDF membrane (Millipore, Billerica, MA, USA) by semidry blotting. The PVDF membrane was incubated with 1000-fold-diluted mouse antibody against His tag for 2 h and then incubated with 2000-fold-diluted HRP-labeled goat anti-mouse IgG (Proteintech, Wuhan, China; 1:2000) for 2 h after washing with phosphate buffer. Signals were visualized by incubation with ECL substrate and development by X-ray (Carestream Health, Rochester, NY, USA). Protein concentrations were determined using a Bio-Rad protein assay (Bio-Rad Laboratories, Hercules, CA, USA) in triplicate against a bovine serum albumin (BSA) standard.

### 2.9. Inhibitory Effect of Recombinant Protein SLAMF7 on Bacterial Survival

The effects of recombinant protein SLAMF7 on survival of *S. aureus* (ATCC) 29213 and *E. coli* (ATCC) 35218 were examined by agar gel diffusion. For agar gel diffusion, bacteria for lawn seeding were grown in liquid LB media. Inocula were spread on solid agar LB plates. Standard paper disks containing 10 µL of recombinant SLAMF7 were placed on the surface of the agar under sterile condition. Instead of the recombinant protein, penicillin and H_2_O were used as the positive and negative controls, respectively. The plates were then cultured overnight at 37 °C. Inhibitory effects were observed on the basis of the inhibitory zone. Similar agar gel diffusion examinations were performed using *E. coli* instead of *S. aureus*. For *E. coli*, kanamycin and H_2_O were used as the positive and negative controls, respectively.

## 3. Results

### 3.1. Study Design and Sample Clustering

**Figure 1:**
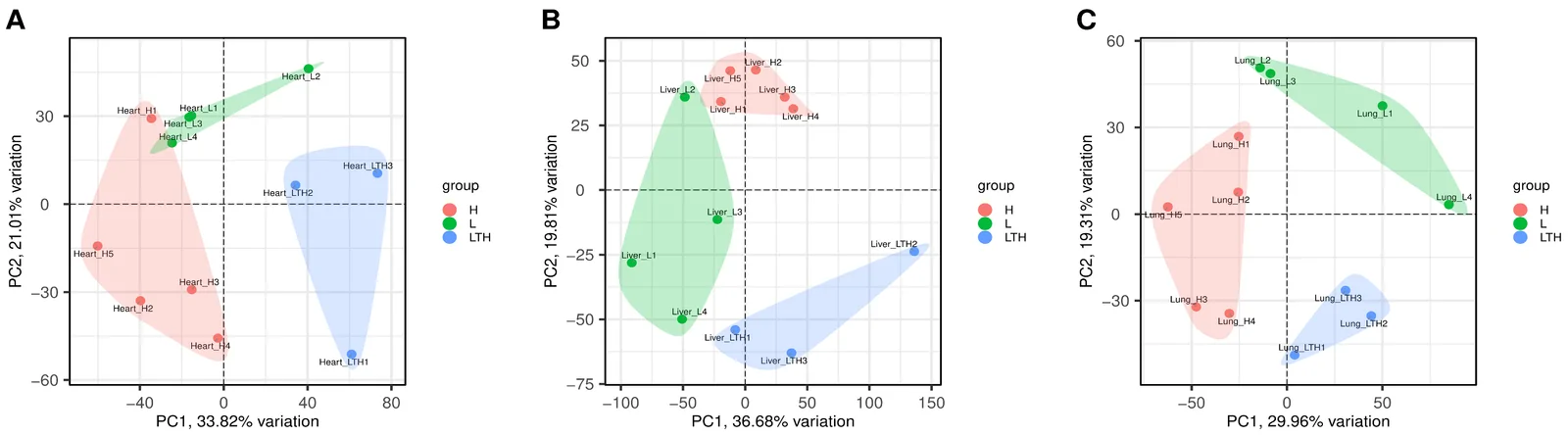
PCA of transcriptomic profiles in the three cattle groups. PCA score plots show H, L, and LTH samples from the (**A**) heart, (**B**) liver, and (**C**) lung. Percentages on the PC1 and PC2 axes indicate the variance explained by each component, and the colored shapes enclose samples from the same group.

Three experimental groups established to simulate distinct adaptive states were used (Figure 1): lowland Holstein cattle (L, *n* = 4) raised in Jinan, China (~100 m); native highland Tibetan cattle (H, *n* = 5) raised in Lhasa, China (~3500 m); and highland-acclimatized Holstein cattle (LTH, *n* = 3) relocated from the lowland to Lhasa and maintained at high altitude for 6 months’ high-altitude acclimatization.

Principal component analysis (PCA) was performed to assess transcriptomic variation among the three groups. PC1 and PC2 together explained 49.09% to 56.49% of the total gene expression variance across the heart, lung, and liver. Within each tissue, samples clustered by group with clear separation and no obvious outliers (Figure 1). These results validated that the transcriptional differences observed among groups were primarily driven by hypoxic adaptive states (short-term physiological acclimatization vs. long-term genetic adaptation) rather than technical variation.

### 3.2. Analysis of Blood Physiological Indicators Related to Hypoxic Adaptation in Cattle

**Figure 2:**
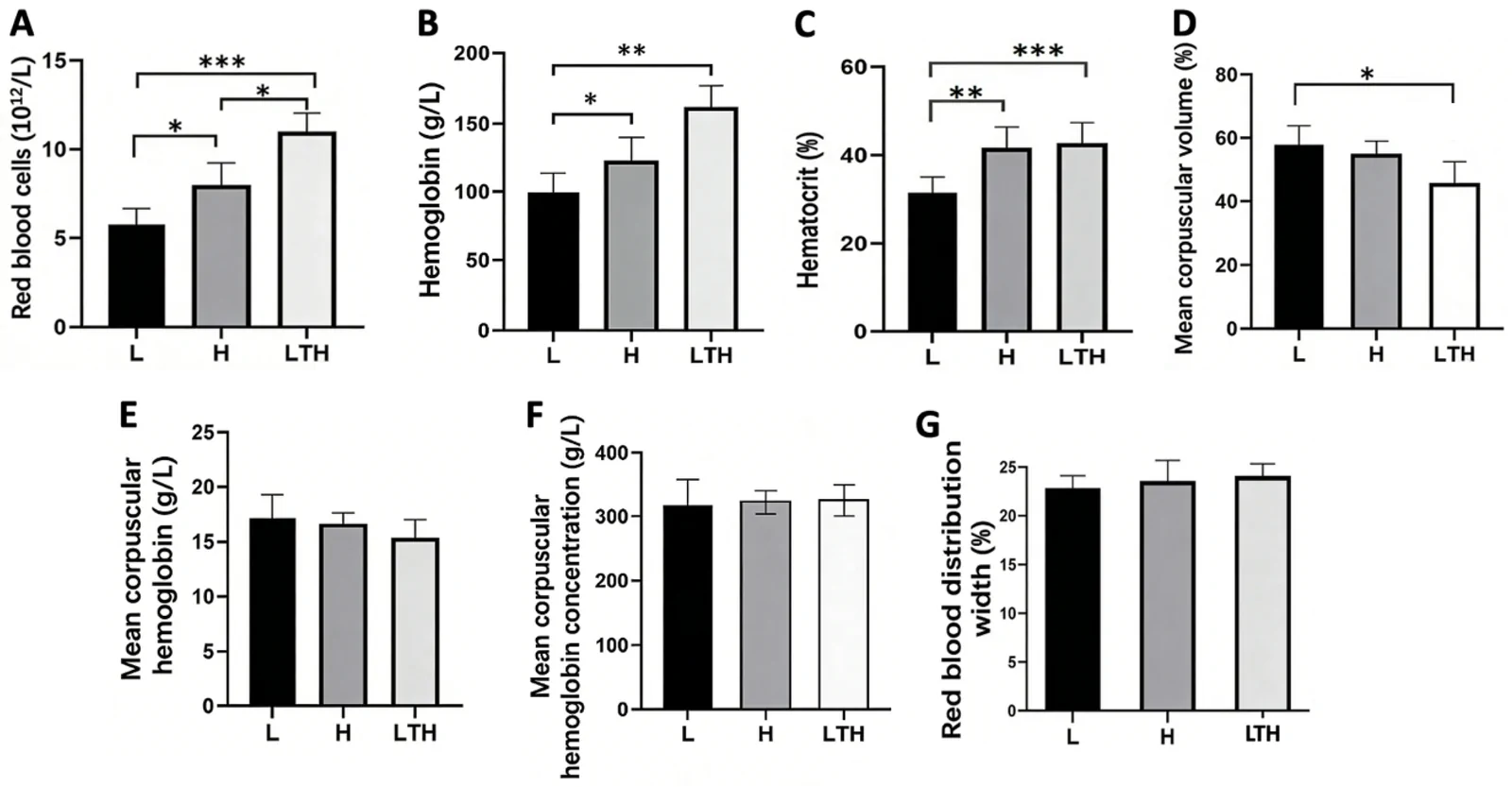
Hematological indices in low-altitude Holstein cattle (L), native highland Tibetan cattle (H), and highland-acclimatized Holstein cattle (LTH). (**A**) Red blood cell count (RBC); (**B**) hemoglobin concentration (HGB); (**C**) hematocrit (HCT); (**D**) mean corpuscular volume (MCV); (**E**) mean corpuscular hemoglobin (MCH); (**F**) mean corpuscular hemoglobin concentration (MCHC); and (**G**) red blood distribution width (RDW). Data are shown as means ± SEM. Pairwise comparisons were performed using two-sided Welch *t*-tests with Bonferroni-adjusted *p* values (* *p* < 0.05, ** *p* < 0.01, and *** *p* < 0.001).

Venous blood samples were collected from three experimental groups to measure hematological and hemorheological indicators. Compared with lowland Holsteins (L), Holsteins acclimatized to the highland for six months (LTH) exhibited significantly increased red blood cell count (RBC) and hemoglobin concentration (HGB), together with decreased hematocrit (HCT), mean corpuscular volume (MCV), and mean corpuscular hemoglobin (MCH) (Figure 2). These results indicated that lowland Holstein cattle enhance hematopoietic capacity to drive adaptive responses to hypoxic high-altitude conditions, and the phenotypic plasticity of these hematological traits is primarily induced by hypoxic stress. In contrast, Tibetan cattle (H) displayed markedly lower RBC and HGB levels compared with LTH cattle, demonstrating that Tibetan cattle have evolved genetic mechanisms for a blunted hypoxic stress response through long-term adaptive evolution. Additionally, no significant differences were observed in mean corpuscular hemoglobin concentration (MCHC) or red blood cell distribution width (RDW) among the three groups (Figure 2), suggesting that cattle do not achieve high-altitude hypoxic adaptation by modulating red blood cell morphology.

**Figure 3:**
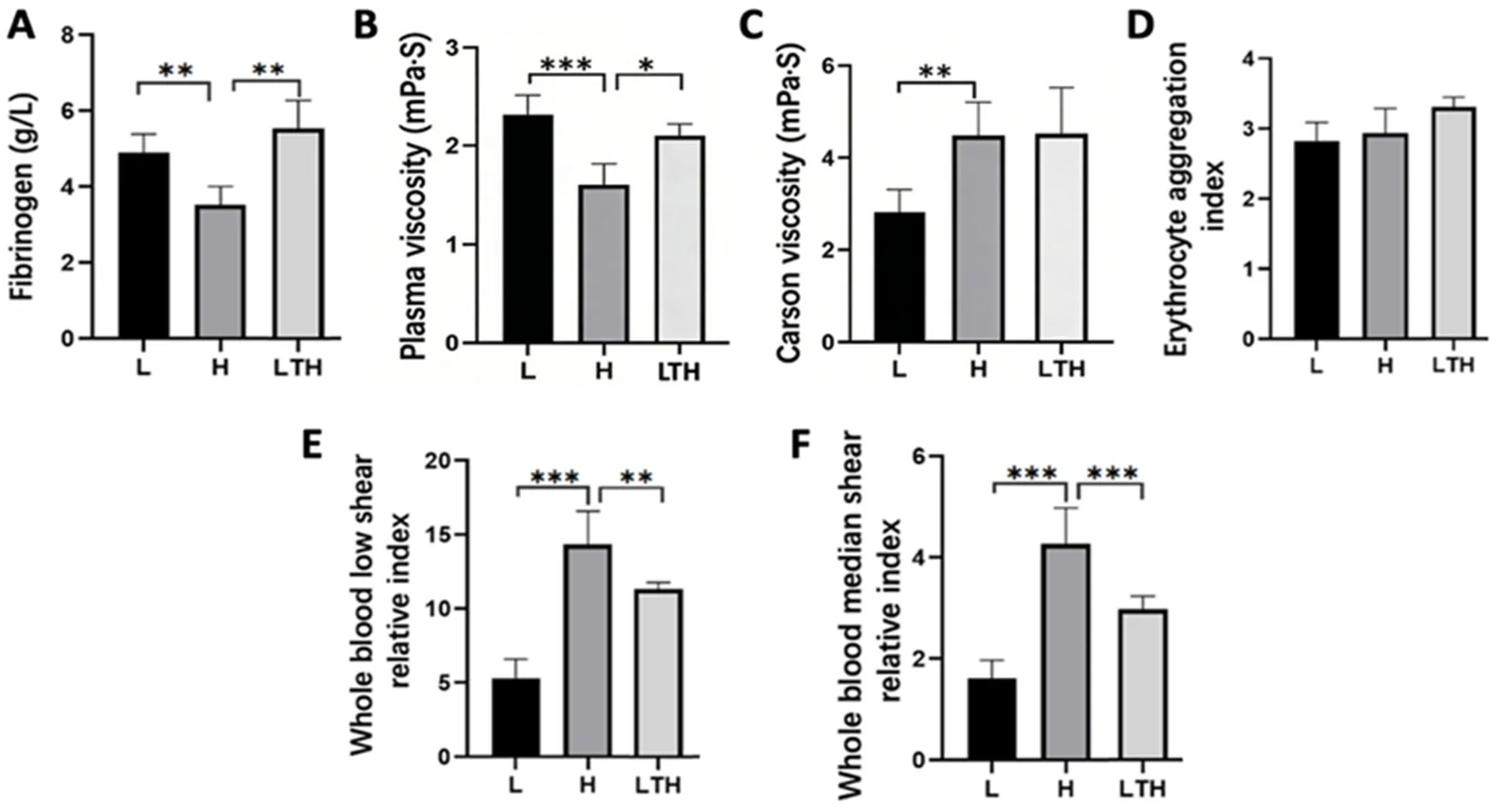
Hemorheological indices in lowland Holstein cattle (L), native highland Tibetan cattle (H), and highland-acclimatized Holstein cattle (LTH). (**A**) Fibrinogen concentration (FIB); (**B**) plasma viscosity (PV); (**C**) Casson viscosity (CV); (**D**) erythrocyte aggregation index; (**E**) whole-blood low-shear relative index; and (**F**) whole-blood median-shear relative index. Data are shown as means ± SEM. Pairwise comparisons were performed using two-sided *t*-tests with Bonferroni correction (* *p* < 0.05, ** *p* < 0.01, and *** *p* < 0.001).

Consistent with the hematological findings, LTH cattle exhibited significantly higher fibrinogen (FIB), plasma viscosity (PV), and Casson viscosity (CV) than lowland Holsteins, indicating hemorheological remodeling after six months at high altitude (Figure 3). Tibetan cattle maintained lower viscosity-related indices than LTH cattle, which constitutes a key physiological mechanism underlying their high-altitude adaptation. The erythrocyte aggregation index did not differ significantly among the three groups.

### 3.3. Identification of Differentially Expressed Genes in Short-Term and Long-Term Hypoxia Adaptation in Cattle

**Figure 4:**
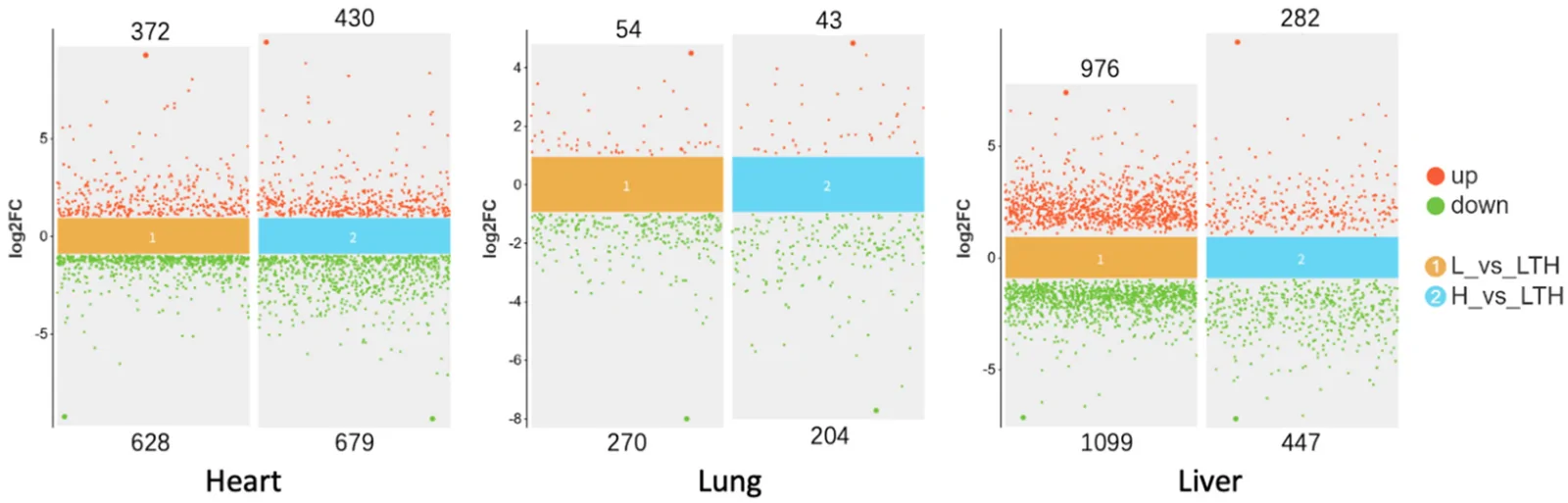
Volcano plot of significantly differential expressed genes. The red dots represent upregulated genes and blue dots downregulated genes. Orange sections represent short-term high-altitude acclimatization (lowland Holstein cattle vs. highland Holstein cattle) and blue long-term high-altitude adaptation (highland Holstein cattle vs. highland Tibetan cattle).

DEGs linked to short-term acclimatization (STA) were identified by comparing native lowland Holstein cattle with highland-acclimatized Holstein cattle. As these groups share an identical genetic background, but inhabit distinct altitudes, the observed DEGs are driven by short-term hypoxic environmental stimuli. Conversely, DEGs related to long-term adaptation (LTA) were defined by comparing highland-acclimatized Holstein cattle with native highland Tibetan cattle, reflecting gene expression alterations from long-term high-altitude exposure and inherent genetic adaptation. Using a threshold for false-discovery rate (FDR) <0.05 and *p* < 0.01, 1000, 324, and 2075 DEGs were detected in the heart, lung, and liver (respectively) for STA, while 1109, 247, and 729 DEGs were identified in the same tissues for LTA. Notably, the lung exhibited fewer DEGs in both acclimatization and adaptation compared to the heart and liver, highlighting distinct organ-specific hypoxia sensitivity (with the lung showing relatively lower responsiveness). Interestingly, the liver harbored fewer DEGs in LTA than in STA (contrasting the heart’s slight increase in LTA-associated DEGs), indicating that the liver may have established a stable transcriptional regulatory state during long-term hypoxic adaptation, reducing the need for extensive gene expression remodeling (Figure 4).

### 3.4. RT-qPCR Validation of RNA-Seq Results

**Figure 5:**
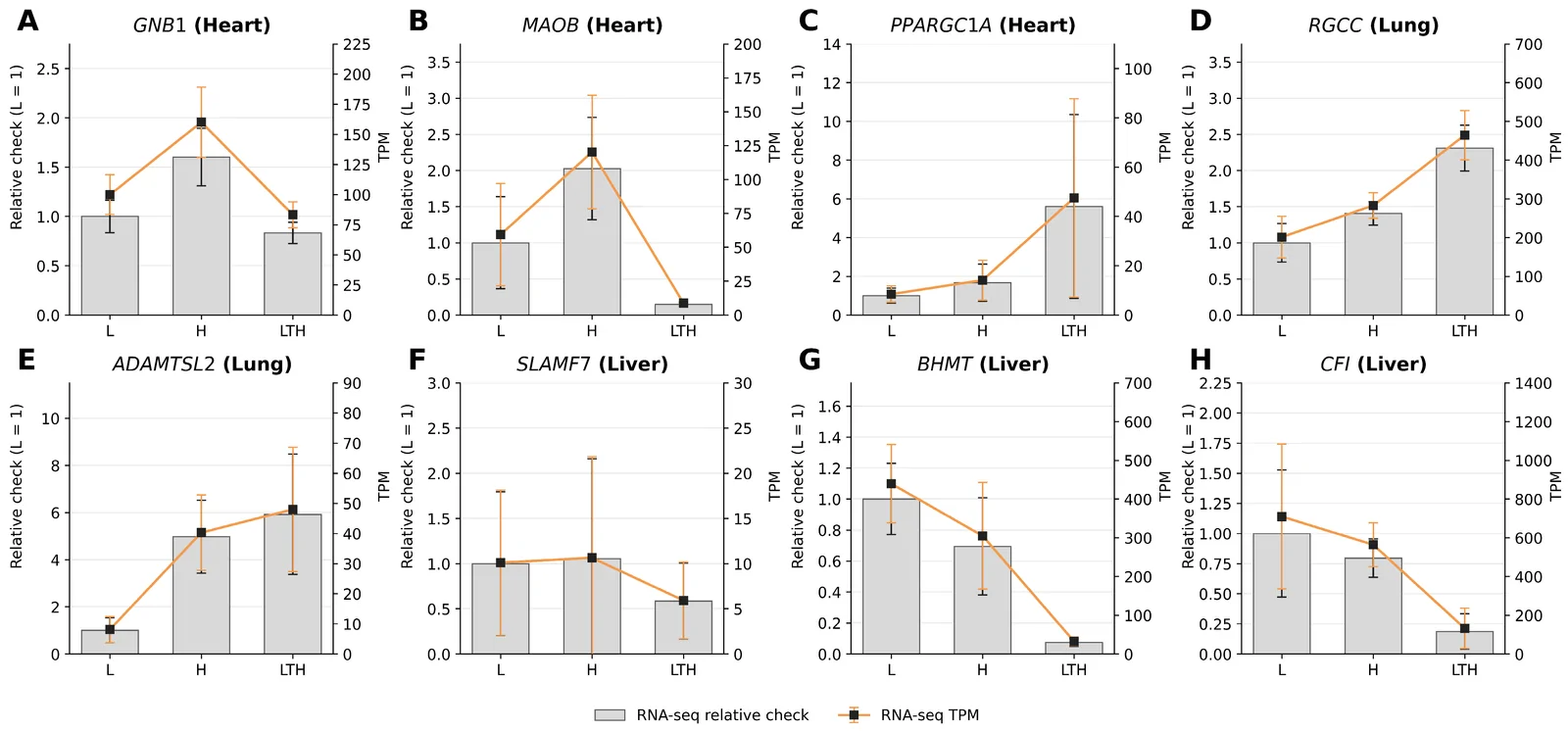
RT-qPCR validation of selected gene expression patterns identified by RNA-seq. Gray bars represent relative expression determined by RT-qPCR (left y-axis), whereas orange lines with black squares represent RNA-seq TPM values (right y-axis). (**A**) *GNB1* in the heart; (**B**) *MAOB* in the heart; (**C**) *PPARGC1A* in the heart; (**D**) *RGCC* in the lung; (**E**) *ADAMTSL2* in the lung; (**F**) *SLAMF7* in the liver; (**G**) *BHMT* in the liver; and (**H**) *CFI* in the liver.

The expression patterns of the eight selected genes determined by RT-qPCR were generally consistent with the RNA-seq TPM trends across the L, H, and LTH groups (Figure 5). This concordance across the heart, lung, and liver supports the reliability of the transcriptomic results.

### 3.5. Key Transcription Genes During Hypoxia Stress Identified by WGCNA

**Figure 6:**
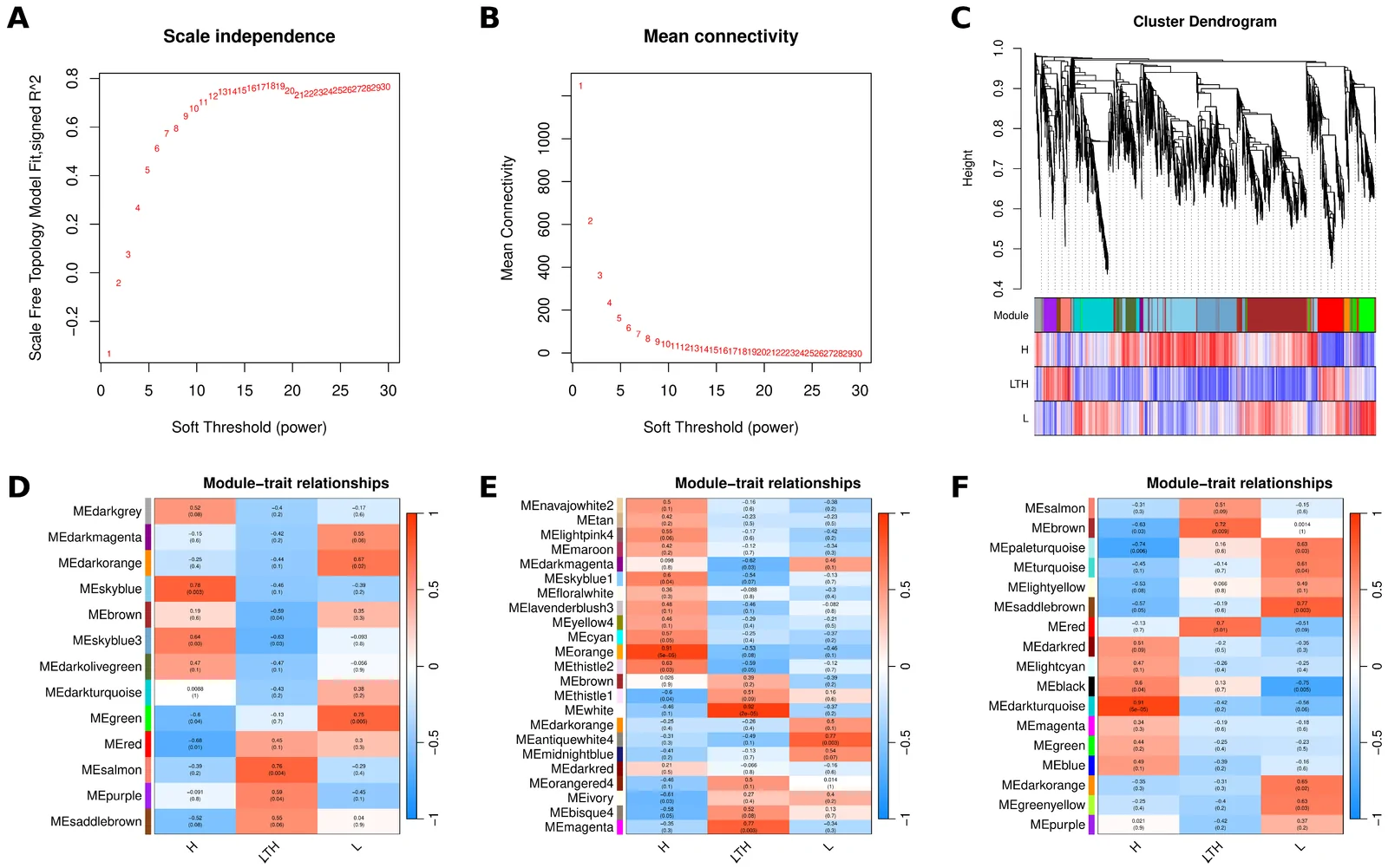
Core results of weighted gene co-expression network analysis. (**A**) Gene cluster dendrogram. Hierarchical clustering tree of genes (**top**) and corresponding module color bar (**bottom**), where different colors represent distinct co-expression modules identified by dynamic tree cutting. (**B**) Scale independence analysis. Scale-free topology fitting index (R^2^) across different soft-thresholding powers (β) used to select the optimal β that satisfied the scale-free network property. (**C**) Mean connectivity analysis. Trend of average gene connectivity with increasing soft-thresholding powers, assisting in confirming the appropriate β parameter. (**D**–**F**) Module–trait relationship heatmaps. Correlations between co-expression modules and target traits. The color intensity (red = positive correlation, blue = negative correlation) represents the correlation coefficient, and values in each cell indicate the correlation coefficient (**upper**) and corresponding *p*-value ((**lower**), in parentheses). ME represents the module eigengene, and the module–trait relationships are shown separately for the heart (**D**), liver (**E**), and lung (**F**).

Weighted gene co-expression network analysis (WGCNA) was performed to characterize tissue-specific gene networks associated with short-term hypoxic acclimatization and long-term genetic adaptation in cattle. To construct a robust scale-free network, a soft-thresholding power of *β* = 7 was chosen, at which the scale-free topology fit index reached >0.6 and relatively high connectivity was maintained (Figure 6A,B). The characteristic values of different modules were significantly correlated with oxygen stimulation, indicating that the genes within these modules may regulate the adaptation of cattle to high-altitude hypoxia through a synergistic effect (Figure 6C). Specifically, in heart tissue, modules MElightblue3 and MEskyblue exhibited positive correlations with long-term adapted Tibetan cattle, while showing negative correlations with short-term acclimatized Holstein cattle (Figure 6D). The distinct divergence suggests that the MElightblue3 and MEskyblue module likely mediates taxon-specific adaptive mechanisms underpinning the long-term high-altitude adaptation of native Tibetan cattle, with potential involvement of genes that regulate cellular homeostasis in hypoxic environments. Similar phenomena also occur in the liver, MEdarkturquoise modules showed the strongest positive correlation with Tibetan cattle (correlation coefficient 0.91), indicating a core role in long-term adaptation (Figure 6E). In lung tissue, multiple modules (MEorange, MEskyblue1, MEcyan) were positively correlated with H and negatively correlated with LTH. Furthermore, the MEwhite module was prominently positively correlated with LTH, highlighting its central role in acute pulmonary hypoxic response (Figure 6F). Collectively, these results uncovered conserved co-expression networks in heart and lung for long-term hypoxia adaptation alongside tissue-specific modules for short-term acclimatization, providing a foundation for subsequent hub gene identification and functional validation.

### 3.6. Functional Enrichment of DEGs in Biological Pathways and Gene Ontologies in Multiple Oxygen-Sensitive Organs

**Figure 7:**
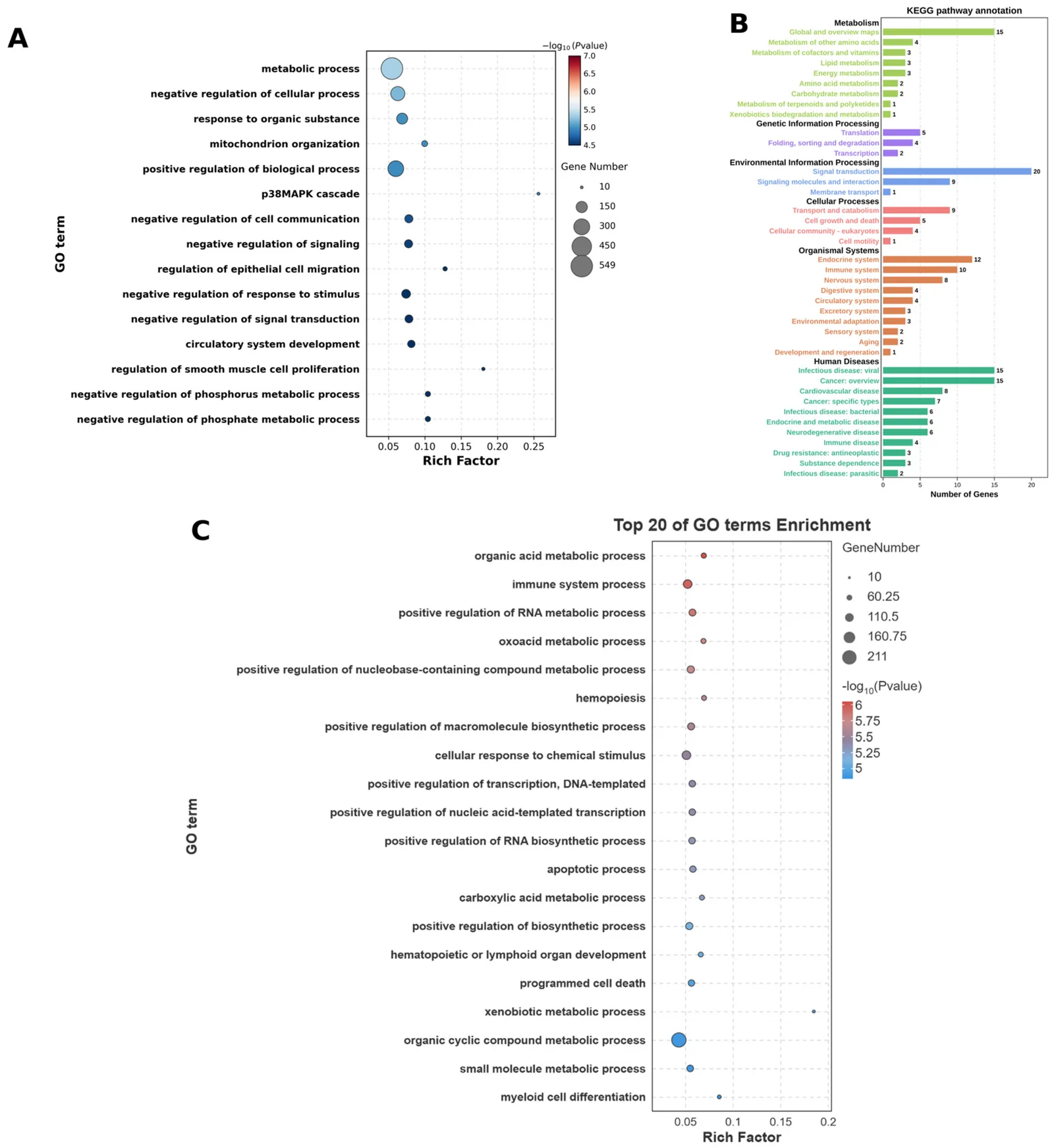
Enrichment analysis of differentially expressed genes in oxygen-sensitive organs. (**A**) Bubble plot of GO functional enrichment (biological process), showing significantly enriched functional entries. Rich factor indicates the enrichment degree, and the color gradient represents −log10(*p*-value) (red for higher significance). (**B**) Bar chart of KEGG pathway enrichment, displaying significantly enriched pathways and the corresponding number of genes across five categories: metabolism, genetic information processing, environmental information processing, cellular processes, and organismal systems. (**C**) Bubble plot of GO functional enrichment (biological process), focusing on core functional entries such as organic acid metabolic process and immune system process, with rich factor and color gradient having the same meanings as in (**A**). In panels (**A**,**C**), bubble size represents the number of enriched genes.

To prioritize candidate biological pathways and processes involved in STA and LTA, we conducted gene set enrichment analysis (GSEA) on genes annotated to the DEGs. We observed that DEGs associated with STA were significantly enriched in metabolic processes across both heart and liver tissues. Specifically, metabolic processes included organic substance metabolism, macromolecule metabolism, and nitrogen compound metabolism, functional categories that are central to regulating energy supply and cellular homeostasis under hypoxic stress (Figure 7A,B). Notably, while this metabolic enrichment pattern was conserved between the two organs, the magnitude of enrichment was more pronounced in the heart: ~1137 heart-derived STA DEGs mapped to metabolic processes (enrichment score = 11.3%) compared to ~890 liver-derived STA DEGs (enrichment score = 9.1%). This observation collectively suggests that the heart tissue prioritizes metabolic reprogramming as its primary adaptive strategy to cope with hypoxic stress, an evolutionarily favorable tactic given the heart’s irreplaceable role in maintaining systemic oxygen delivery and its high energy demand even under oxygen limitation. Compared to STA, the LTA DEGs categories were enriched in immune system process and hemopoiesis pathways in liver, indicating the multiple roles of liver tissue in environmental adaptation (Figure 7C). This finding is in line with the transcriptomic study of yaks at high altitudes, in which the expression levels of calcium channels decrease, whereas innate immunity becomes more active [16,17]. In addition, the lung exhibits a different pattern from the other two organs, especially in the pattern of long-term adaptation, LTA DEGs in lung were enriched in cell circulation, including nucleic acid metabolic process, RNA metabolic process and nucleobase-containing compound metabolic process, suggesting that cell circulation play a major role in LTA. This result aligns with observations from the patterns of pulmonary arterial hypertension, caused by excessive proliferation of lung cells [18].

### 3.7. Validation of Key Pathway Genes Expression for Hypoxia Stimulation

**Figure 8:**
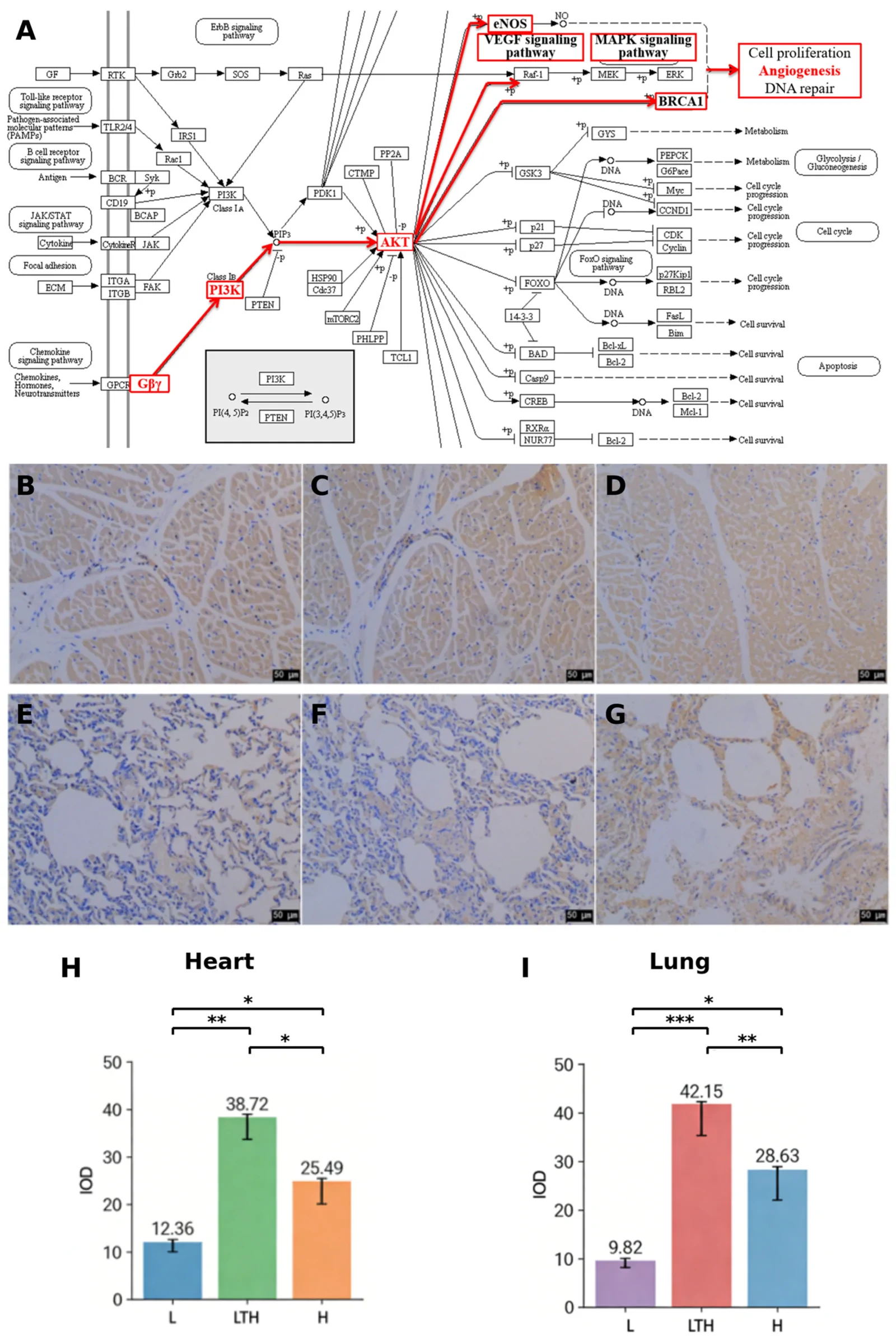
Immunohistochemical staining of *GNB1* gene expression in heart and lung tissue. (**A**) KEGG pathway of *GNB1*. Permission to use this KEGG pathway map image was kindly granted by Kanehisa Laboratories. (**B**) Immunohistochemical staining of heart tissue with GNB1 in lowland Holstein cattle. (**C**) Immunohistochemical staining of heart tissue with GNB1 in highland Holstein cattle. (**D**) Immunohistochemical staining of heart tissue with GNB1 in highland Tibetan cattle. (**E**) Immunohistochemical staining of lung tissue with GNB1 in lowland Holstein cattle. (**F**) Immunohistochemical staining of lung tissue with GNB1 in highland Holstein cattle. (**G**) Immunohistochemical staining of lung tissue with GNB1 in highland Tibetan cattle. Brown or tan particles are immune-positive cells. (**H**) Quantification of GNB1 immunoreactivity in heart tissue. (**I**) Quantification of GNB1 immunoreactivity in lung tissue. Data are presented as means ± SEM. Pairwise comparisons were performed using two-sided *t*-tests with Bonferroni correction; * *p* < 0.05, ** *p* < 0.01, and *** *p* < 0.001.

The G protein subunit beta 1 (*GNB1*) gene encodes the β subunit of heterotrimeric G proteins, which are composed of *α*, *β* and *γ* subunits. G proteins modulate multiple blood pressure regulatory systems including the nervous system, renin–angiotensin system and endothelin system by mediating downstream receptor-coupled signaling [19]. WGCNA results revealed that *GNB1* was localized in the significant modules across all three cattle groups (|cor| > 0.7, *p* < 0.01). KEGG pathway enrichment analysis further identified GNB1 as a component of the PI3K-AKT signaling pathway (Figure 8A; KEGG database [20]), which modulates angiogenesis and thereby contributes to high-altitude hypoxic adaptation in cattle [21]. To validate the protein-level expression pattern of GNB1—a key hypoxia-responsive gene and core regulatory factor of the oxygen-sensing pathway—immunohistochemical (IHC) staining was performed to detect GNB1 expression in the heart and lung tissues of cattle from the three experimental groups. The results demonstrated significant intergroup differences in both the localization and expression levels of GNB1-positive signals. In heart tissue (Figure 8B–D), GNB1-positive signals were localized in the cytoplasm and nucleus of cardiomyocytes. Quantitative IOD analysis (Figure 8H) showed that Group L exhibited weak GNB1 expression with sparse positive granules. Following short-term high-altitude acclimatization, Group LTH showed a significant enhancement in GNB1 expression, which was 3.13-fold higher than that in Group L (*p* < 0.01). Group H displayed moderate GNB1 expression, which was significantly lower than Group LTH (*p* < 0.05), but significantly higher than Group L. In lung tissue, GNB1-positive signals were predominantly distributed in alveolar epithelial cells, bronchial epithelial cells and vascular endothelial cells (Figure 8E–G), with an expression pattern consistent with that in heart tissue. Quantitative IOD analysis (Figure 8I) showed that Group L had the weakest GNB1 expression. Group LTH showed a dramatic upregulation of GNB1 expression, which was 4.29-fold higher than that in Group L (*p* < 0.001). Group H exhibited moderate GNB1 expression, which was significantly reduced compared with Group LTH (*p* < 0.01), but significantly higher than Group L (*p* < 0.05). Notably, GNB1-positive cells in Group H were concentrated at the alveolar-capillary interface, whereas the positive signals in Group LTH showed a diffuse distribution pattern.

### 3.8. SLAMF7 Enhances Antimicrobial Immunity as a Key Mediator of High-Altitude Adaptation in Cattle

**Figure 9:**
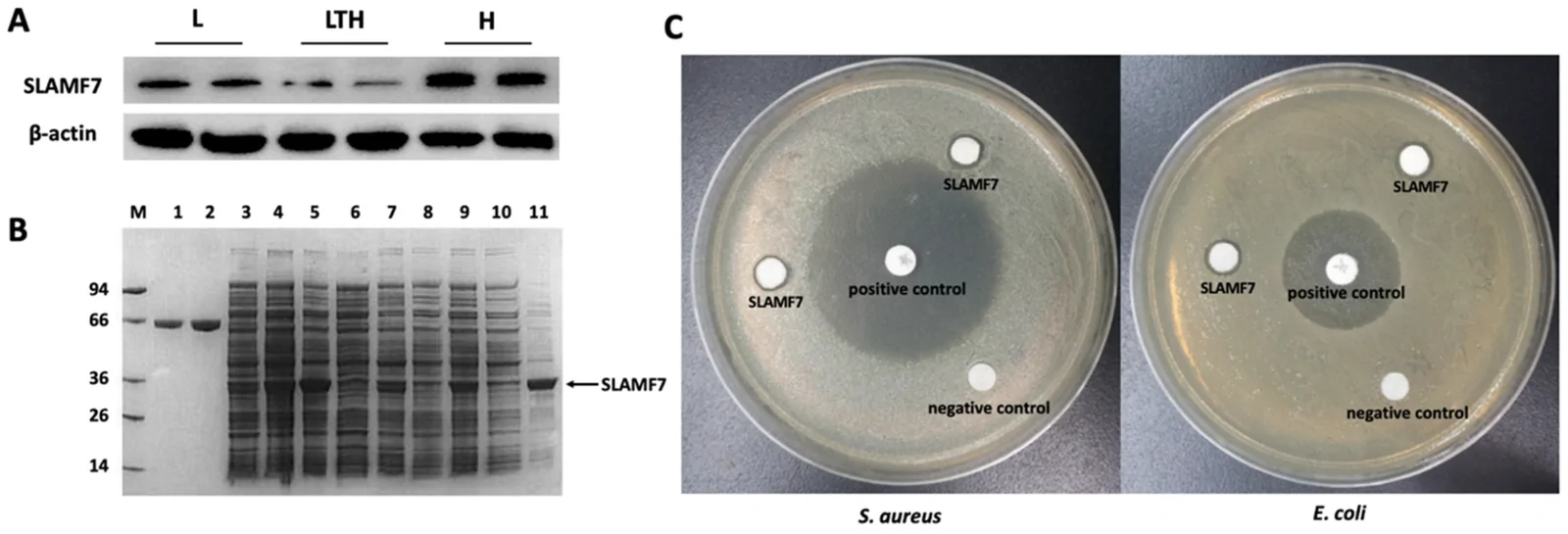
Expression validation and functional characterization of SLAMF7. (**A**) Western blot analysis of SLAMF7 protein expression in liver tissues from three cattle groups. (**B**) SDS-PAGE analysis of prokaryotic expression and purification of recombinant SLAMF7 protein. Lane M: protein molecular weight marker; lane 11: purified recombinant SLAMF7 protein (~35 kDa, consistent with the predicted molecular weight. (**C**) Antimicrobial activity assay of recombinant SLAMF7 protein against *Staphylococcus aureus* (**left**) and *Escherichia coli* (**right**) using the agar diffusion method. The positive control (ampicillin) and negative control (PBS) were set as references. The original Western blot images are summarized in Figure S1.

To further clarify the immune-related mechanisms underlying short- and long-term high-altitude adaptation, we performed expression validation and functional characterization of the candidate gene signaling lymphocyte activation molecule family 7 (*SLAMF7*), which is a member of the SLAM immunoreceptor family that plays an important role in innate and adaptive immunity [22,23]. Western blot analysis demonstrated that SLAMF7 protein expression was significantly elevated in the liver tissue of native highland Tibetan cattle (H group) compared to both lowland Holstein cattle (L group) and highland-acclimatized Holstein cattle (LTH group), with the lowest expression levels detected in the LTH group (Figure 9A). Subsequently, the recombinant SLAMF7 protein was successfully expressed in BL21 *Escherichia coli* strain and purified to homogeneity, yielding a band of approximately 33.65 kDa that matched the predicted molecular weight (Figure 9B). Functional assays via the agar diffusion method revealed that recombinant SLAMF7 exhibited antibacterial activity against both *Staphylococcus aureus* (Gram-positive) and *Escherichia coli* (Gram-negative), as evidenced by clear inhibition zones (Figure 9C). Collectively, these findings suggest that *SLAMF7* serves as a key functional gene mediating long-term high-altitude adaptation in cattle. The upregulated expression and direct antimicrobial activity collectively enhance immune defense and antibacterial capacity in highland environments, thereby representing a critical adaptive strategy for survival under hypoxic stress.

## 4. Discussion

In this study, we used a unique experimental design to delineate physiological and molecular adaptations attributable to short-term high-altitude exposure and those resulting from long-term high-altitude exposure. We set a six-month plateau acclimatization duration to differentiate transient acute hypoxic stress from stable long-term physiological and transcriptional remodeling. A representative multi-temporal multi-omics ruminant study on translocated Hu sheep utilized an eight-month rearing endpoint for the identical research purpose [24]. Longitudinal transcriptomic investigations focused on dairy cattle further verified that 5–6 months of continuous plateau feeding enables RBC, HGB, blood viscosity and multi-organ transcriptional networks to reach a stable equilibrium, eliminating the volatile stress fluctuations observed within the first three months of hypoxia exposure [25]. Holsteins after six months at high altitude showed increased RBC and HGB levels, indicating a compensatory response that may enhance oxygen-carrying capacity. In contrast, Tibetan cattle maintained lower RBC and HGB levels than highland-acclimatized Holsteins, resembling the restrained erythropoietic phenotype reported in Tibetan human populations and other high-altitude-adapted species [26,27,28]. Rather than maximizing erythropoiesis, this strategy may balance oxygen transport and blood fluidity, thereby reducing the hematological cost of chronic hypoxia [29,30,31,32]. Changes in fibrinogen (FIB), plasma viscosity (PV), and Casson viscosity (CV) further indicated that acclimatization involved hemorheological remodeling in addition to hematopoietic compensation. Although this remodeling may support physiological compensation, sustained increases in viscosity-related indices could increase circulatory resistance. However, these indices did not change uniformly among the three groups, suggesting that long-term adaptation cannot be interpreted simply as a reduction in blood viscosity. Moreover, the absence of significant differences in erythrocyte aggregation contrasts with findings in some yak studies [2], indicating species-specific contributions of cell aggregation and shear-dependent viscosity to hypoxia tolerance. A prominent limitation of this study is the lack of direct measurement of peripheral blood oxygen saturation (SpO_2_) in experimental cattle, and thus we draw relatively conservative conclusions regarding hypoxic responses based solely on blood hematological and hemorheological indices. Overall, short-term acclimatization appears to rely primarily on hematological compensation accompanied by rheological costs, whereas long-term adaptation favors a more restrained balance between oxygen-carrying capacity and blood fluidity.

At the molecular level, our findings revealed distinct tissue-specific transcriptional responses in the heart, liver, and lung, consistent with the physiological functions of these organic. In the heart, DEGs identified during six-month acclimatization were primarily enriched in organic substance and macromolecule metabolic pathways, whereas the broader transcriptional response associated with long-term adaptation suggested sustained metabolic reprogramming. Because the myocardium has a continuous and high demand for ATP, this metabolic adjustment may help maintain energy supply, macromolecular turnover, and contractile activity under hypoxia. Prioritizing metabolic rather than extensive immune remodeling may also limit inflammation-related impairment of cardiac function. The liver exhibited a different response pattern, with more extensive transcriptional changes during six-month acclimatization, but fewer and more selective changes during long-term adaptation. This pattern suggests that the liver initially mounts a broad stress response and subsequently develops a more stable regulatory state. Notably, the enrichment of immune and hematopoietic pathways during long-term adaptation may reflect the liver’s combined roles in nutrient metabolism, detoxification, and immune surveillance of portal blood. Similar shifts from broad stress responses to more selective long-term regulation have been reported in other organisms [33,34,35], although breed background, diet, and microbial exposure may also contribute to the hepatic immune signature. The lung showed fewer transcriptional changes than the heart and liver, with enrichment mainly in nucleic acid and RNA metabolic pathways, suggesting that pulmonary adaptation may rely more on cellular renewal and transcriptional homeostasis than on extensive pathway-level remodeling. In addition, the localization and differential expression of GNB1 in cardiomyocytes and at the alveolar–capillary interface, together with its association with PI3K/AKT signaling, suggest a potential role in angiogenic and oxygen-sensitive responses [19,20]. However, these findings support an association rather than direct activation of the PI3K/AKT pathway or a causal role for GNB1 in hypoxic adaptation.

Consistent with the enrichment of immune-related pathways in the liver, *SLAMF7* emerged as a candidate associated with the hepatic immune response of Tibetan cattle. As an activating receptor expressed on NK cells, CD8+ T cells, monocytes, and dendritic cells, SLAMF7 regulates immune-cell adhesion and activation [36,37] and may also limit excessive pro-inflammatory cytokine production [25,38]. In the present study, SLAMF7 expression was significantly elevated in the liver of Tibetan cattle, and recombinant SLAMF7 inhibited both *Staphylococcus aureus* and *Escherichia coli*. These findings suggest that SLAMF7 may contribute to hepatic immune surveillance and antibacterial defense in harsh high-altitude environments. However, its antibacterial activity does not establish a hypoxia-specific function, and the observed expression differences may also be influenced by breed background, diet, microbiota, pathogen exposure, or general environmental stress. Therefore, *SLAMF7* should be considered a candidate contributor to high-altitude immune resilience rather than a confirmed key mediator of hypoxic adaptation. Together with the tissue-specific transcriptional patterns and GNB1 expression, these findings support a multi-organ response model in which the heart, liver, and lung make distinct, but coordinated contributions to high-altitude adaptation.

## 5. Conclusions

This study integrated hematological, hemorheological, and multi-organ transcriptomic data to distinguish six-month high-altitude acclimatization in Holstein cattle from long-term adaptation in Tibetan cattle. Highland-acclimatized Holsteins exhibited increased RBC and HGB levels together with changes in FIB, PV, and CV, indicating enhanced hematological compensation accompanied by potential rheological costs. In contrast, Tibetan cattle maintained a more restrained erythropoietic phenotype, suggesting a different balance between oxygen-carrying capacity and blood fluidity. Transcriptomic analyses further revealed organ-specific responses, characterized by cardiac metabolic remodeling, a hepatic shift toward immune homeostasis, and comparatively limited pulmonary transcriptional changes. Pathway analysis and immunohistochemistry identified GNB1 as a candidate associated with PI3K/AKT-related angiogenic responses, whereas hepatic SLAMF7 expression and recombinant-protein antibacterial activity suggested a potential contribution to high-altitude immune resilience. However, the current evidence supports association rather than direct causation or hypoxia specificity for these candidate genes. Overall, six-month acclimatization and long-term adaptation represent distinct physiological and molecular strategies rather than a simple linear transition. Further studies using larger cohorts, paired physiological and transcriptomic measurements, broader gene-expression validation, and controlled functional experiments are required to verify the proposed mechanisms.

## Data Availability

The datasets generated and/or analyzed during the current study are available in the Genome Sequence Archive (GSA) repository, with the accession number CRA068915 (https://ngdc.cncb.ac.cn/gsa/browse/CRA068915) (accessed on 24 April 2026).

## Supplementary Materials

The following supporting information can be downloaded at https://www.mdpi.com/article/10.3390/biology15171474/s1. Table S1: Transcript abundance (TPM) across all RNA-seq samples; Table S2: Differential expression statistics for the L-versus-LTH and H-versus-LTH comparisons in heart, lung, and liver; Table S3: Primer sequences used for RT-qPCR validation; Table S4: Genes in highly correlated co-expression modules identified by WGCNA in heart; Table S5: Genes in highly correlated co-expression modules identified by WGCNA in lung; Table S6: Genes in highly correlated co-expression modules identified by WGCNA in liver; Table S7: Gene Ontology enrichment results for the L-versus-LTH and H-versus-LTH comparisons in heart, lung, and liver. Figure S1: The original Western blot images.
